# Assessing whether child and parent reports of the KINDL questionnaire measure the same constructs of quality of life in children with attention-deficit hyperactivity disorder

**DOI:** 10.1186/s12955-020-01649-w

**Published:** 2021-01-15

**Authors:** Marzieh Alamolhoda, Mohammad Farjami, Zahra Bagheri, Ahmad Ghanizadeh, Peyman Jafari

**Affiliations:** 1 Department of Biostatistics, Shiraz University of Medical Sciences, Shiraz, Iran; 2 Research Center for Psychiatry and Behavioral Sciences, Shiraz University of Medical Sciences, Shiraz, Iran

**Keywords:** Quality of life, Attention deficit hyperactivity disorder, Parents, Children, Questionnaire

## Abstract

**Background:**

Discrepancy between child self-report and parent proxy-report has long been documented in the health-related quality of life (HRQoL) measurement of children with chronic health conditions. This study aims to assess whether child and parent reports of the Kinder Lebensqualität fragebogen (KINDL) questionnaire measure the same construct of HRQoL in children with attention-deficit hyperactivity disorders (ADHD).

**Methods:**

Participants were 122 Iranian children with ADHD and 127 of their parents, who completed the child and parent reports of the KINDL, respectively. Internal consistency of the child and parent reports were assessed by Cronbach's alpha. The intra-class correlation (ICC) coefficient and factor analysis were applied to assess whether the child self-report and the parent proxy-report measured the same construct of HRQoL. Additionally, convergent and discriminant validity were assessed using the Spearman correlation.

**Results:**

The results of factor analysis revealed that the child self-report and parent proxy-report measure two different aspects of HRQoL. Moreover, both versions of the KINDL instrument showed excellent convergent and discriminant validity. The internal consistency was close to or greater than 0.7 for all domains of both child and parent reports.

**Conclusions:**

Although the child self-report and the parent proxy-report of the Persian version of the KINDL have good psychometric properties, they are not interchangeable. This finding indicates that Iranian children with ADHD and their parents evaluate children's HRQoL from their own viewpoints.

## Background

Attention-deficit hyperactivity disorder (ADHD) is the most common neuropsychiatric disorder during childhood, affecting about 5% of the pediatric population [1, 2]. A study on a community sample of Iranian children reported that about 10.1% of children have some symptoms of ADHD [3]. As compared with other physical and mental health disorders, ADHD can seriously impair a child's quality of life (QoL) [4]. Previous studies have shown that the health-related quality of life (HRQoL) in children with ADHD is lower than that of children with chronic diseases such as asthma and cancer [5–7]. Children with ADHD suffer from symptoms of inattention, hyperactivity, and impulsivity. Such impairments can be reflected in various domains of a child’s HRQoL, including academic performance, school behavior, peer relations, and physical, psychological, cognitive, and family functioning [8].

In recent years, a number of pediatric QoL questionnaires have been developed to evaluate children’s HRQoL according to both child and parent reports. Although child self-report is considered to be an accurate measure for assessing HRQoL, the parent-proxy report provides complementary information regarding children’s HRQoL [9, 10]. A systematic review of the published QoL studies in children and adolescents with ADHD has shown that most of the reviewed studies have only used parents as informants and not asked the children themselves about their QoL [4]. The previous studies have shown that children with ADHD and their parents have different perspectives regarding children’s HRQoL, and children generally tend to rate their own HRQoL better than their parents [1, 4, 11–13]. In most of the aforementioned studies, there is a consistent discrepancy across the child-self report and parent-proxy report on the children’s HRQoL [1, 12, 13]. It is likely that the observed disagreement reflects a wider perceptual issue across child and parent reports in general. Moreover, the discrepancies between self and proxy-reports could vary substantially across different samples and from one measure to another. Therefore, assessing the agreement between the child and parent reports on children’s HRQoL and also choosing the most appropriate HRQoL questionnaire is an important objective in clinical research.

According to a systematic review, the Child Health Questionnaire (CHQ), and the Pediatric Quality of Life Inventory (PedsQL™ 4.0) are two well-known instruments that have been frequently used to evaluate HRQoL in children with ADHD [4, 11]. Although these generic instruments are designed to measure different aspects of QoL in children and adolescents, a systematic review has shown that pediatric QoL instruments do not exactly reflect the World Health Organization (WHO) definitions of QoL [14]. In general, the PedsQL™ 4.0 questionnaire is designed to assess a wide definition of functioning, disability, and health (FDH), the KIDSCREEN is an appropriate instrument to assess HRQoL, and the CHQ and the KINDL are developed to measure FDH with some HRQoL features. Therefore, it is important to be aware of what kind of tools the researchers want to measure relative to standard definitions and their intended purpose.

The KINDL is a reliable and valid instrument [15] that is used nationally and internationally in different languages and countries [15–21]. A distinguishing feature of the KINDL, as compared with other pediatric QoL instruments, is the inclusion of both positively and negatively worded items in a single questionnaire which can reduce ceiling and floor effects as well as response bias. According to Lin et al. [22, 23], the existence of positively and negatively worded effects, which may be a threat to the construct validity of the questionnaire, can be adjusted by using methods such as a multitrait-multimethod approach along with confirmatory factor analysis. Previous studies have shown that there is a significant association between self-esteem and QoL when the KINDL is used to measure QoL compared with other generic QoL questionnaires for children [24, 25].

Although the KINDL is one of the most widely accepted tools for assessing children's HRQoL in clinical and non-clinical samples [15], there are a limited number of studies that have used this questionnaire for measuring the HRQoL in ADHD children [26, 27]. As far as we know, there is no study available to assess the agreement between child self-report and parent proxy-report for a sample of children diagnosed with ADHD. Hence, in the present study, we intend to determine whether the child and parent reports of the KINDL questionnaire measure the same constructs of HRQoL in children with ADHD. Moreover, as an essential prerequisite for assessing agreement or disagreement between self and proxy-ratings, the psychometric properties of the KINDL will be evaluated in Iranian children with ADHD.

## Method

### Participants and instruments

The study sample was composed of children and adolescents with ADHD and also their parents refereeing to a tertiary care clinic of Shiraz University of Medical Sciences, Shiraz, Iran. ADHD diagnosis was made according to DSM-IV criteria with children and their parents [28], using the Kiddie Schedule for Affective Disorders and Schizophrenia– (K-SADS) Persian version [29]. The children and their parents were asked to complete a set of documents containing child self-report and parent proxy-report of the KINDL. Moreover, the written informed consent and the explanations of the protection of personal information were obtained from the parents. The study protocol was approved by the Ethics Committee of Shiraz University of Medical Sciences.

The Persian version of the child self-report and parent proxy-report of the KINDL, which had been previously translated and validated in Iranian school children [17], was completed by 122 children (74.6% boys, 25.4% girls) with ADHD and 127 of their parents, respectively. The mean (SD) age of the Iranian children with ADHD was 10.40 (2.10). Due to the sample size limitation, we combined the Kid-KINDL (for 8 to12-year-old children) and the Kiddo-KINDL (for 13 to 16-year-old adolescents) versions to obtain a larger sample size. The Kid and Kiddo versions of the KINDL include 24 items in six domains: physical well-being, emotional well-being, self-esteem, family, friends, and school [15]. The participants responded to the items on a 5-point Likert scale (0 = never, 1 = seldom, 2 = sometimes, 3 = often, and 4 = all the time). All subscales were then transformed to a 0–100 score, with higher scores representing better HRQoL.

### Statistical analysis

The reliability and validity of the KINDL were evaluated by using the traditional classical test theory (CTT) approach. Cronbach’s alpha coefficient was used to assess the reliability of the KINDL subscales. Internal consistency was considered satisfactory if the coefficient was at least 0.7. Moreover, the percentages of responding to each item on the minimum and maximum scales were measured to determine floor and ceiling effects. Substantial floor or ceiling effects occur when more than 40% of respondents choose “not at all” or “very much” categories [30]. Two independent sample tests were used to compare the mean of subscale scores between children and their parents. Convergent and discriminant validity was checked using Spearman correlation. The value of a correlation coefficient of greater than 0.40 between an item and its own hypothesized scale provides evidence of convergent validity. Discriminant validity is supported whenever a correlation between an item and its hypothesized scale is higher than its correlation with the other scales. A scaling success rate is counted if the item to own-scale correlation is significantly higher than the correlations of the item to another scale [31].

The exploratory factor analysis with varimax rotation was used to assess the construct validity of self and proxy-ratings of the KINDL. It also was used to test whether the child and parent-reports measure the same construct of HRQoL. Moreover, the Intra-class correlation (ICC) coefficient was calculated for 91 child-parent dyads to assess the agreement between child and parent reports of the KINDL measure. ICC coefficient calculates the ratio of the variance between respondents to the within-respondents. The strength of agreement between the two measures was classified as excellent (greater than 0.80), good (0.61 to 0.80), moderate (0.41 to 0.60), and poor (0.40 or less) agreements [32]. Statistical analyses were conducted using SPSS (SPSS Inc. Released in 2007. SPSS for Windows, Version 16.0. Chicago, IL, USA). *p *Values of less than 0.05 were found to have statistically significant.

## Results

The overall Cronbach’s alpha coefficients in this study were 0.85 and 0.81 for the child self-report and parent proxy-report of the Persian version of the KINDL questionnaire, respectively. The results of the internal consistency, floor and ceiling effects, and also the convergent and discriminant validity of the child and parent reports are presented in Table 1. The mean scores of the subscales ranged from 58.61 (Self-esteem) to 70.39 (Physical well-being) for the child self-report and 49.11 (School) to 65.65 (Physical well-being) for the parent proxy-report. As shown in Table 1, Iranian children with ADHA rated their HRQoL significantly higher than their parents in all domains (*p *values < 0.05). No substantial floor or ceiling effects were detected in all of the subscales on both versions of the KINDL, except for the emotional well-being subscale in the child self-report which had a ceiling effect greater than 40%. The range of internal consistency was between 0.70 and 0.76 for all subscales of the KINDL, except for physical well-being (0.67) and emotional well-being (0.67) subscales in the child self-report and the parent proxy-reports, respectively. Moreover, the scaling success rates for the convergent and discriminant validity were 100% in all domains of the self and proxy-ratings. According to these findings, both versions had excellent convergent and discriminant validity.

**Table 1:** Item scaling test: convergent and discriminant validity for KINDL subscales in children and their parents

	Items	Mean ± SD^a^	Floor effect (%)	Ceiling effect (%)	Cronbach’$\alpha$	Convergent validity^b^		Discriminant validity^c^	
						Range of correlation	Scaling success (%)	Range of correlation	Scaling success (%)
*Child self-reports*									
Physical well-being	4	70.39 ± 21.21	6.1	37.7	0.67	0.63–0.75	4/4 (100)	0.006–0.49	20/20 (100)
Emotional well-being	4	68.90 ± 23.29	6.3	40.2	0.72	0.67–0.77	4/4 (100)	0.03–0.50	20/20 (100)
Self-esteem	4	58.61 ± 26.00	13.9	29.7	0.74	0.71–0.83	4/4 (100)	0.001–0.33	20/20 (100)
Family	4	66.34 ± 23.60	8.6	35.7	0.70	0.68–0.76	4/4 (100)	0.06–0.38	20/20 (100)
Friends	4	66.44 ± 24.44	10.6	37.7	0.70	0.66–0.81	4/4 (100)	0.06–0.45	20/20 (100)
School	4	63.52 ± 26.35	13.1	38.1	0.73	0.73–0.76	4/4 (100)	0.01–0.37	20/20 (100)
Total score	24	65.70 ± 15.53							
*Parent proxy-reports*									
Physical well-being	4	65.65 ± 19.67^*^	3.3	26.0	0.70	0.61–0.79	4/4 (100)	0.03–0.34	20/20 (100)
Emotional well-being	4	59.94 ± 19.31^*^	4.1	20.9	0.67	0.62–0.79	4/4 (100)	0.005–0.31	20/20 (100)
Self-esteem	4	52.26 ± 21.77^*^	8.3	13.2	0.76	0.69–0.82	4/4 (100)	0.03–0.32	20/20 (100)
Family	4	53.44 ± 20.59^*^	8.1	14.2	0.76	0.68–0.76	4/4 (100)	0.01–0.44	20/20 (100)
Friends	4	58.02 ± 20.27^*^	7.1	17.7	0.70	0.64–0.82	4/4 (100)	0.01–0.34	20/20 (100)
School	4	49.11 ± 22.50^*^	14.6	14.6	0.73	0.69–0.77	4/4 (100)	0.002–0.31	20/20 (100)
Total score	24	56.41 ± 12.14^*^							

^a^standard deviation

^b^Number of correlation between items and hypothesized scale corrected for overlap ≥ 0.4/ total number of convergent validity tests

^c^Number of convergent correlations significantly higher than discriminant correlations/Total number of correlations

^*^Indicates that the corresponding subscales between child and parent reports are statistically significant (*p* values < 0.05)

The results of the exploratory factor analysis of the self and proxy-reports of the KINDL are presented in Table 2. In general, 60.0% and 59.7% of the proportions of variance were explained by the first six factors in the child and parent reports, respectively. In the parent proxy-report, all of the items had strong correlations with their own subscales. In the child report, all of the items that should be in the emotional, self-esteem, family, friends, and school subscales were clearly loaded on their own domains. However, three of the four items in the physical well-being subscale had strong correlations with the emotional well-being subscale.

**Table 2:** Factor loadings^a^ of six factor solution of the KINDL

	Child self-reports						Parent proxy-reports					
	F1	F2	F3	F4	F5	F6	F1	F2	F3	F4	F5	F6
*Physical well-being*												
1. I felt ill	0.15	**0.60**	− 0.15	0.25	0.19	− 0.15	**0.78**	0.11	− 0.11	0.18	0.20	0.06
2. I was in pain	0.50	**0.42**	− 0.10	0.17	0.11	− 0.10	**0.77**	0.09	− 0.01	0.05	0.05	0.04
3. I was tired and worn-out	0.09	**0.75**	0.06	0.00	0.03	0.06	**0.73**	0.21	0.12	0.13	0.00	0.04
4. I felt strong and full of energy	0.73	**0.28**	0.14	− 0.16	0.11	0.14	**0.52**	− 0.09	0.46	− 0.14	0.01	− 0.02
*Emotional well-being*												
1. I had fun and laughed a lot	0.27	**0.45**	0.34	0.10	0.22	0.60	0.13	**0.51**	0.43	0.17	0.06	− 0.24
2. I was bored	− 0.02	**0.77**	0.10	0.06	0.20	0.26	0.01	**0.64**	− 0.17	− 0.22	0.25	− 0.08
3. I felt alone	0.15	**0.60**	− 0.15	0.32	0.14	− 0.02	0.16	**0.78**	0.04	0.14	0.02	0.02
4. I felt scared or unsure of myself	0.09	**0.64**	− 0.05	0.38	− 0.06	− 0.01	0.14	**0.74**	0.15	0.13	− 0.08	0.19
Self-esteem												
1. I was proud of myself	0.14	− 0.02	**0.84**	0.14	− 0.01	0.12	0.00	0.05	**0.80**	0.03	0.09	− 0.03
2. I felt on top of the world	− 0.10	− 0.30	**0.69**	0.08	0.07	− 0.10	0.06	− 0.06	**0.73**	− 0.17	− 0.15	0.12
3. I felt pleased with myself	0.20	0.10	**0.61**	0.17	0.26	− 0.04	− 0.04	0.08	**0.77**	0.19	0.16	0.16
4. I had lots of good ideas	− 0.07	0.05	**0.68**	0.04	0.07	0.25	0.03	0.10	**0.54**	− 0.07	0.22	0.29
Family												
1. I got on well with my parents	0.01	0.11	0.32	**0.67**	0.17	0.17	0.03	− 0.16	0.20	**0.66**	0.32	0.17
2. I felt fine at home	0.35	0.12	0.30	**0.62**	0.12	0.02	0.08	0.10	0.42	**0.48**	0.43	0.00
3. We quarreled at home	0.06	0.19	0.02	**0.61**	0.02	0.20	0.04	0.17	− 0.07	**0.69**	0.14	0.16
4. I felt restricted by my parents	− 0.01	0.23	− 0.06	**0.64**	0.16	0.14	0.20	0.05	− 0.12	**0.74**	− 0.09	0.03
*Friends*												
1. I did things together with my friends	− 0.06	− 0.02	0.26	− 0.01	**0.70**	0.33	0.02	− 0.01	0.10	− 0.19	**0.72**	0.09
2. I was a "success" with my friends	0.16	0.03	0.21	0.16	**0.72**	− 0.11	0.11	0.07	− 0.01	0.20	**0.79**	0.01
3. I got along well with my friends	0.10	0.18	0.03	0.18	**0.78**	− 0.03	0.10	− 0.08	− 0.03	0.23	**0.68**	0.08
4. I felt different from other children	0.09	0.49	− 0.23	0.08	**0.52**	0.18	0.01	0.18	0.12	0.07	**0.50**	0.01
*School*												
1. doing the schoolwork was easy	0.58	0.02	0.13	0.24	0.00	**0.55**	0.04	− 0.06	0.23	0.00	0.37	**0.63**
2. I found school interesting	0.62	0.04	− 0.03	0.26	0.15	**0.49**	− 0.05	− 0.04	0.25	− 0.01	0.41	**0.63**
3. I worried about my future	0.06	0.11	0.04	0.17	0.10	**0.72**	0.03	0.12	− 0.02	0.25	0.00	**0.73**
4. I worried about getting bad marks	− 0.04	0.08	0.11	0.13	− 0.01	**0.76**	0.10	0.00	0.04	0.08	− 0.16	**0.78**

Extraction method: principal component with varimax rotation

F1: Physical functioning, F2: emotional well-being, F3: self-esteem, F4:family, F5: friends, F6: school, factor loadings greater than 0.4 have been bolded

The factor analysis was also used to test whether the self- and proxy- reports measure the same constructs of children's HRQoL or not. The Kaiser–Meyer–Olkin measure of Sampling Adequacy (KMO = 0.68) and Bartlett’s Test of Sphericity (χ^2^ (66) = 270.93, *p* < 0.001) indicated that the data were adequate for conducting the exploratory factor analysis. As shown in Table 3, the first factor extracted includes all subscales of the child report, and the second factor encompasses all subscales of the parent report. Moreover, according to Table 4, the relatively low levels of agreement were observed between the corresponding subscales of the child and parent reports (ICC ranges from − 0.02 to 0.21).

**Table 3:** Factor loadings^1^ of the KINDL in child self-report and parent proxy-report

Scales	Factor 1	Factor 2
*Child self-reports*		
Physical well-being	0.21	**0.51**
Emotional well-being	0.16	**0.48**
Self-esteem	− 0.16	**0.62**
Family	0.15	**0.66**
Friends	0.09	**0.61**
School	− 0.17	**0.62**
*Parent proxy-reports*		
Physical well-being	**0.66**	0.05
Emotional well-being	**0.76**	0.04
Self-esteem	**0.32**	0.12
Family	**0.78**	− 0.06
Friends	**0.68**	0.03
School	**0.59**	0.07

1: Extraction method: principal component with varimax rotation

Items belonging to the postulated scales are shown by bold numbers

**Table 4:** Agreement between child and parent ratings using the ICC coefficients

	ICC	95% CI	*p *Value
Physical well-being	0.16	− 0.05–0.35	0.07
Emotional well-being	0.21	0.001–0.39	0.02
Self-esteem	− 0.02	− 0.22–0.19	0.56
Family	0.21	0.002–0.39	0.02
Friends	0.11	− 0.09–0.31	0.14
School	0.08	− 0.13–0.28	0.22
Total scores	0.10	− 0.11–0.30	0.18

CI: confidence interval; ICC: intra-class correlation

## Discussion

To the best of our knowledge, this is the first study that has evaluated the psychometric properties of the KINDL questionnaire for a sample of children diagnosed with ADHD and their parents. The findings of the present study indicate that the Persian versions of the child and parent reports of the KINDL are reliable and valid instruments for measuring HRQoL when applied to a sample of Iranian children with ADHD. Moreover, exploratory factor analysis revealed that the Persian version of the self and proxy-ratings of the KINDL encompasses six underlying constructs, including physical well-being, emotional well-being, self-esteem, family, friends, and school functioning, except for emotional well-being in child report. This finding indicated that the Persian version of the self and proxy-ratings of the KINDL measured the construct that it was intended to measure. The results of Table 3 showed that for the children diagnosed with ADHD, the items ''I felt ill'' and ''I was tired and worn-out'' are clearly associated with emotional well-being construct rather than physical well-being. It can be attributed to the children's perception of the meanings of sentences or phrases. Sometimes it may be required to adopt the items when we used them in different languages and cultural settings. It seems that, in Iranian ADHD children, phrases such as "I felt" frequently referred more to mood positions rather than physical responses. Children did not have a clear perception of the concept of the items in physical and emotional well-being factors and could not distinguish between the items in these two constructs. These results are in line with the findings of other studies in Iranian diabetic and ADHD children [6, 33]. However, the parents have been able to differentiate QoL subscales rather than did their children.

Our results revealed that the HRQoL scores of children with ADHD were statistically higher than that of their parents, which was in line with the findings of previous studies [12, 34–38]. Moreover, our findings were similar to the previous study in Iran, which reported that children with ADHD evaluated their QoL higher than their parents across all domains of the PedsQL™ 4.0 [6]. The results of the study by Marques in a sample of children with ADHD showed that children rated their own QoL higher than their parents for the emotional, school, and psychosocial functioning [1]. On the other hand, Varni and Burwinkle reported that the parent and child ratings were similar across all domains of PedsQL™ 4.0 [7].

An interesting finding of the present study was that the same domains in the self and proxy reports were weakly correlated. Accordingly, the exploratory factor analysis extracted two different HRQoL constructs: one included all of the domains of the child self-reports and the other corresponding to all of the domains of the parent proxy-reports. Moreover, the relatively low ICC coefficients of the pairwise subscales of the child and parent reports of the KINDL confirmed the results of the factor analysis. These findings provide sufficient evidence to conclude that the child self-report and parent proxy-report of the KINDL measure two different constructs of HRQoL in children with ADHD, and hence are not interchangeable. This finding indicates that children with ADHD and their parents have a different perception of the HRQoL concept. This is consistent with the two previous studies which reported that child and parent ratings of the HRQoL of children with chronic conditions could not simply be substituted [39, 40]. Moreover, our results were consistent with previous studies, where a poor parent–child agreement was reported in HRQoL of children with ADHD [13, 41].

It should be noted that the type of disease and its severity could affect the agreement between child and parent reports. Although the imperfect agreement between child and parent reports regarding the child’s HRQoL has been previously documented in QoL literature [40], future studies should examine the agreement between child-self reports and parent-proxy reports of the KINDL in other clinical samples, languages, and cultures [24, 25, 42]. In addition, as child-parent agreement can be greatly affected by the measure used, further studies are also needed to investigate whether the child and parent reports of the other pediatric HRQoL instruments measure the same or different construct of QoL in children with ADHD. However, if we intend to draw a general conclusion linking the findings of the present research and the previous ones, it would be that parent-reports should be used to provide complementary information about the child's HRQoL, especially when children are not able to evaluate their own QoL due to their health conditions [42].

The present study has a number of limitations that need to be mentioned. First, this study was conducted in a limited homogenous sample of ADHD children in the south of Iran. Accordingly, the results do not have external validity to generalize to all ADHD children. Second, although the present study showed that parents of children with ADHD reported the child’s HRQoL significantly lower than their children in all domains of the KINDL questionnaire, these results should be interpreted with caution. Indeed, assessing the agreement across child and parent ratings regarding the child’s HRQoL is currently in transition, from comparing means or ICC coefficients to adopting new methods such as differential item functioning (DIF) analysis. It means that we should be confident that the items comprising the KINDL questionnaire operate consistently between the children and their parents. When DIF exists, the comparison of the HRQoL subscale scores across children and their parents may be incorrect. Hence, in future studies, DIF analysis should be conducted to evaluate whether children with ADHD and their parents perceive the meaning of the items in the KINDL questionnaire consistently [17].

## Conclusions

The Persian version of the child self-report and parent proxy reports of the KINDL questionnaire can be used as a reliable and valid instrument to assess HRQoL in Iranian children with ADHD. Furthermore, the results of the present study revealed that the child and parent reports of the KINDL questionnaire measure distinct concepts related to HRQoL. Therefore, there is a need to apply simultaneously the child and parent reports of the KINDL for measuring QoL in children with ADHD.

## Data Availability

The datasets used and/or analyzed during the current study available from the corresponding author on reasonable request.

## Abbreviations

ADHD: Attention-deficit hyperactivity disorder
HRQoL: Health related quality of life
KINDL: Kinder Lebensqualität fragebogen
QoL: Quality of life
CHQ: Child health questionnaire
PedsQL™ 4.0: Pediatric quality of life inventory
WHO: World health organization
FDH: Functioning, disability and health
K-SADS: Kiddie schedule for affective disorders and schizophrenia
ICC: Intra-class correlation
